# Retraction: Transcriptome and Multivariable Data Analysis of *Corynebacterium glutamicum* under Different Dissolved Oxygen Conditions in Bioreactors

**DOI:** 10.1371/journal.pone.0205785

**Published:** 2018-10-11

**Authors:** The PLOS ONE Editors

Following publication, concerns were raised regarding overlap between this article [1] and a previous article in the Journal of Industrial Microbiology & Biotechnology [2], which was not cited and discussed:

“Comparative analysis of the *Corynebacterium glutamicum* transcriptome in response to changes in dissolved oxygen levels”

Authors: Xiuxia Liu, Sun Yang, Fen Wang, Xiaofeng Dai, Yankun Yang, Zhonghu Bai

The corresponding author, Dr. Zhonghu Bai, has stated that while both studies use the same transcriptome data, the research questions and methodology were different. Specifically, the *PLOS ONE* article focuses on the energy-related genes affected by DOT levels, and a new method (MVDA) was used to analyze the transcriptome data and metabolism data to identify genes which can regulate the balance of NADH/NAD^+^ and affect ATP production. The prior article reported on the influence of different DOT concentrations on genetic regulation and metabolism through transcriptomic analysis.

Following evaluation of the issues identified and discussion with a member of our Editorial Board, the *PLOS ONE* Editors have determined that although the two papers have different focuses and the MVDA analysis represents a new contribution, there is substantial overlap in the reported data. A number of figures and tables (Fig 1, 2, 7, S2, S3, and Table 2, S3, S4, S5) presented in the *PLOS ONE* article and in the Supporting Information contain overlapping data or are similar or identical to those in the previously published work.

The *PLOS ONE* Editors issue a retraction of the article owing to the concerns about significant redundancy and the lack of disclosure of a closely related study using the same data set under consideration elsewhere at the time of submission, which is a requirement under *PLOS ONE* editorial policies.

YS, WWG, FW, FP, YKY, XFD, XXL, and ZHB agreed with the retraction.
